# Use of volume-targeted non-invasive bilevel positive airway pressure ventilation in a patient with amyotrophic lateral sclerosis*,**

**DOI:** 10.1590/S1806-37132014000400013

**Published:** 2014

**Authors:** Montserrat Diaz-Abad, John Edward Brown

**Affiliations:** Sleep Disorders Center, University of Maryland; and Assistant Professor of Medicine. University of Maryland School of Medicine, Baltimore, MD, USA; Sleep Disorders Center, University of Maryland; and Assistant Professor of Medicine. University of Maryland School of Medicine, Baltimore, MD, USA

**Keywords:** Amyotrophic lateral sclerosis, Respiratory insufficiency, Hypoventilation, Intermittent positive-pressure ventilation, Sleep

## Abstract

Amyotrophic lateral sclerosis (ALS) is a progressive neurodegenerative disease in which most patients die of respiratory failure. Although volume-targeted non-invasive bilevel positive airway pressure (BPAP) ventilation has been studied in patients with chronic respiratory failure of various etiologies, its use in ALS has not been reported. We present the case of a 66-year-old woman with ALS and respiratory failure treated with volume-targeted BPAP ventilation for 15 weeks. Weekly data downloads showed that disease progression was associated with increased respiratory muscle weakness, decreased spontaneous breathing, and increased use of non-invasive positive pressure ventilation, whereas tidal volume and minute ventilation remained relatively constant.

## Introduction

Amyotrophic lateral sclerosis (ALS) is a progressive neurodegenerative disease. Most ALS patients die of respiratory failure due to progressive respiratory muscle weakness, with a median survival of less than 2 years after diagnosis.^(^ 1 ^)^ Non-invasive positive pressure ventilation (NPPV) prolongs and improves the quality of life of patients with ALS.^(^ 2 ^)^ The use of volume-targeted, non-invasive bilevel positive airway pressure (BPAP) ventilation, in spontaneous-timed (ST) mode with adjustment of inspiratory pressure to provide an estimated target tidal volume (V_T_), has been studied in patients with chronic respiratory failure of various etiologies. ^(^ 3 ^-^ 8 ^)^ However, we are unaware of any reports of its use in a patient with ALS. 

We report the case of a patient with ALS with rapidly progressive disease and hypercapnic respiratory failure who was treated at home with volume-targeted BPAP ST mode ventilation. Weekly monitoring of downloaded ventilator data was accompanied by routine clinical follow-up.

## Case report

A 66-year-old woman without a significant past medical history and with a body mass index of 23.4 kg/m^2^ presented with mild bulbar symptoms followed by right foot drop. At 11 months after symptom onset, she was diagnosed with ALS. At that time, FVC was 2.22 L (79% of predicted) and MIP was −28 cmH_2_O (40% of predicted). Her ALS Functional Rating Scale (ALSFRS) score was 34 (out of 40) with a bulbar component score of 10 (out of 12), denoting mild impairment. Her Pittsburgh Sleep Quality Index (PSQI) score was 8 (out of 21), which is consistent with poor sleep quality, whereas her Epworth Sleepiness Scale (ESS) score was 4 (out of 24), indicating no evidence of excessive daytime sleepiness. 

At 4 months of follow-up, marked disease progression was evident, with worsening bulbar symptoms and fatigue, as were new conversational dyspnea, orthopnea, and nonrestorative sleep. Her pulmonary function and functional status had declined-FVC, 1.58 L (57% of predicted); MIP, −25 cmH_2_O (36% of predicted)-and her ALSFRS score was 28 with a bulbar component score of 8. Sleep scores were relatively unchanged (PSQI, 7; ESS, 4). An arterial blood gas could not be obtained after two attempts. Gastrostomy and NPPV were recommended. The patient requested further confirmatory testing prior to these interventions, and overnight in-laboratory polysomnography was scheduled for the following week.

Polysomnography revealed sleep hypoventilation. Three weeks later, volume-targeted BPAP ST ventilation titration (Average Volume-Assured Pressure Support; Philips-Respironics, Murrayville, PA, USA) was performed using a full face mask, per patient preference (Table 1). The patient could not tolerate the target V_T_ (8 mL/kg). Therefore, the final settings were V_T_ at 320 mL (6 mL/kg), inspiratory positive airway pressure at 8-15 cmH_2_O, expiratory positive airway pressure at 6 cmH_2_O (increased for flow limitation), and inspiratory time at 1.5 s, with a backup rate of 12 breaths/min. One week later, the patient returned to the clinic with continued worsening of bulbar symptoms and weakness, using a walker, and reporting dyspnea on minimal exertion. Her FVC was 1.05 L (38% of predicted), with an MIP of −19 cmH_2_O (27% of predicted) and a PaCO_2_ of 53 mmHg. Her ALSFRS score was 26, with a bulbar component score of 6, her PSQI score was 17, and her ESS score was 7. Nocturnal NPPV was started with polysomnography settings and a backup rate of 14 breaths/min.

**Table 1:** Sleep study data in a patient with amyotrophic lateral sclerosis.

Parameter	Type of study^a^	
	PSG	AVAPS
Total sleep time, min	250	116
Sleep efficiency, %	55	32
Sleep latency, min	58	113
Total wake time, min (%)	203 (45)	242 (65)
Stage 1, min (%)	5 (1)	15 (4)
Stage 2, min (%)	175 (39)	99 (27)
Stage 3, min (%)	71 (16)	15 (4)
REM, min (%)	0 (0)	0 (0)
Wake after sleep onset, min	143	31
Arousal index, events/h	18	6
Spontaneous arousals, n	67	11
Periodic limb movement index, events/h	1	0
Apnea hypopnea index, events/h	0	0
Mean nocturnal SpO_2_, %	95	97
Minimum SpO_2_, %	93	94
Baseline ETCO_2_, mmHg	46	47-54
Maximum ETCO_2_, mmHg	57	57
ETCO_2_ > 50, min	227	121
Baseline RR, breaths/min	-	24
Final ETCO_2_, mmHg	-	35-45
Final RR, breaths/min	-	12-14

PSG : polysomnography

**AVAPS:** : average volume-assured pressure support

REM : rapid-eye-movement sleep

**ETCO2:** : end-tidal CO2

a The patient performed both studies recumbent at approximately 45°.

**Figure 1:**
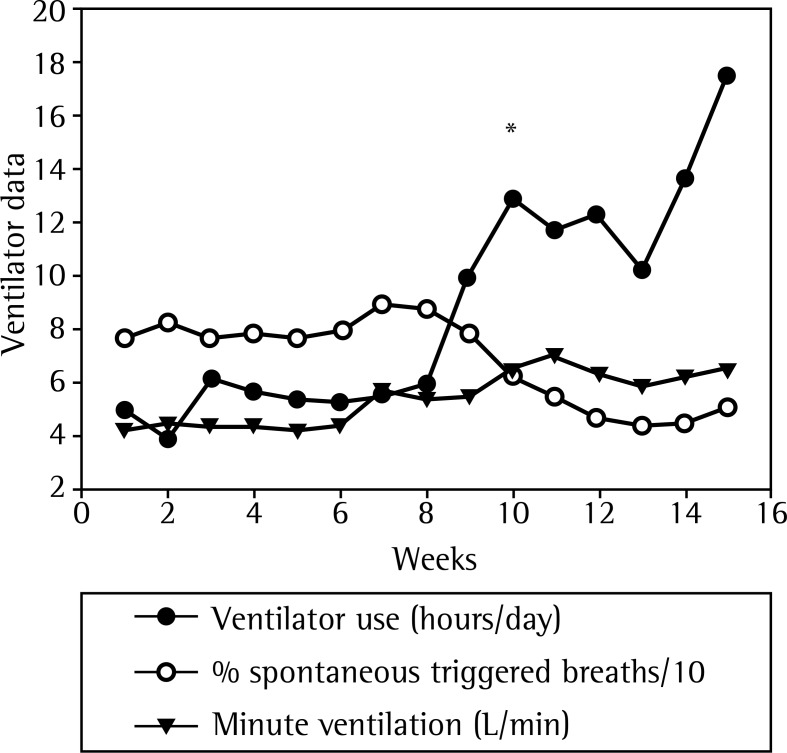
Ventilator data for a 15-week period in an amyotrophic lateral sclerosis patient on bilevel positive airway pressure ventilation. Note the increased duration of daily use of ventilation with decreased ability to trigger breaths spontaneously (i.e., increased reliance on timed ventilator-delivered breaths) over time. As can be seen, minute ventilation remained relatively constant. *Ventilator support increased between weeks 8 and 9; week 10 reflects this increase for the first complete week.

A gastrostomy tube was inserted under radiological guidance, and the patient started home hospice, with no plans to return to the clinic. Seven weeks after starting NPPV, she was contacted to adjust settings based on symptoms and downloaded data (Table 2 and Figure 1), and the patient decided to come to the clinic for a short visit to discuss her worsening dyspnea. She had mild dyspnea at rest and required a wheelchair for mobility. Nocturnal NPPV, which was used every night, helped ease breathing, allowing her to sleep better and longer. She had recently developed a mask leak due to weight loss. At that time, FVC was 1.01 L (36% of predicted), MIP was −15 cmH_2_O (21% of predicted) and PaCO_2_ was 55 mmHg. Settings were adjusted to V_T_ at 370 mL (7 mL/kg), inspiratory positive airway pressure at 10-17 cmH_2_O, and inspiratory time at 1.2 s, with a backup rate of 18 breaths/min. Intermittent daytime NPPV use and a new mask fitting were recommended. Contact with the patient (via telephone and e-mail) was maintained, and the changes were well tolerated. At 6 weeks after her last visit, she was again contacted to adjust settings but declined to make further changes. Shortly thereafter, she died of progressive respiratory failure. 

**Table 2:** Weekly ventilator data downloads for an amyotrophic lateral sclerosis patient on bilevel positive airway pressure ventilation.

Variable	Week														
	1	2	3	4	5	6	7	8	9	10^a^	11	12	13	14	15
V_E_, L/min	4.3	4.5	4.4	4.4	4.3	4.5	5.8	5.4	5.5	6.5	7.1	6.4	5.9	6.3	6.5
V_T_, mL	271	267	266	259	265	259	295	273	252	310	354	346	336	336	338
Trigger, %	77	82	77	78	76	80	90	87	78	63	55	47	43	46	50
Daily use, h	5.0	3.9	6.1	5.7	5.4	5.3	5.6	6.0	10.1	12.9	11.8	12.4	10.3	13.7	17.6
Use ≥ 4 h/day, %	71	43	100	100	86	86	86	100	100	86	100	100	43	100	100
RR, breaths/min	19	19	20	19	20	20	22	23	25	24	22	20	20	21	22
AHI, events/h	15.2	19.4	25.8	21.6	19.1	23.2	5.2	13.4	22.3	26.8	7.7	15.1	11.4	5.5	12.7
Leak, L/min	40	40	41	38	42	38	36	39	41	38	38	36	36	35	36
IPAP, cmH_2_O	11.9	11.9	12.9	12.8	12.5	12.6	10.9	11.8	13.5	15.0	13.8	13.5	14.2	14.0	14.5

VE : minute ventilation

VT : tidal volume

**Trigger:** : patient-triggered (spontaneous) breaths

**Daily use:** : device use per 24-h period

Use = 4 h/day: days on which the device was used for = 4 h/day

AHI : apnea-hypopnea index

Leak : total mask leak

IPAP : inspiratory positive airway pressure.

a Ventilator support increased between weeks 8 and 9; week 10 reflects this increase for the first complete week

## Discussion

We have presented the case of a patient with ALS treated for chronic respiratory failure with volume-targeted BPAP ST mode ventilation for 15 weeks, in whom the use of weekly monitoring of ventilator data in addition to routine care provided useful information for management of respiratory failure. Disease progression was associated with worsening respiratory muscle weakness, a decrease in spontaneous breathing, and increased use of NPPV, although V_T_ and minute ventilation (V_E_) remained relatively constant. To our knowledge, the use of this mode of NPPV has not been reported in ALS. 

Among patients with ALS, the progression of the disease is relatively rapid but varies.^(^ 9 ^)^ Therefore, serial NPPV pressure adjustments may be required in order to compensate for declining respiratory muscle strength and increasing hypercapnia. ^(^ 10 ^)^ An NPPV mode with an inspiratory pressure range to maintain a target V_T_, rather than a fixed pressure, might reduce the frequency of required adjustments over time in some patients. This feature might also be of benefit in the short term, such as during sleep, when patients with diaphragmatic weakness are vulnerable to worsening hypoventilation, especially during rapid-eye-movement sleep. Ambogrio et al. showed that, in comparison with traditional BPAP ST mode ventilation, volume-targeted BPAP ST mode ventilation was better able to maintain V_E_ (by maintaining V_T_) during sleep in patients with obesity hypoventilation syndrome.^(^ 4 ^)^

The built-in software of NPPV devices is proprietary, and, in the absence of independent validation, the data provided on many parameters should be considered as indicators of trends without any guarantee as to linearity of the estimations provided.^(^ 11 ^)^ Despite this limitation, the available data can provide valuable information for patient management. Studies involving remote monitoring of NPPV compliance data in patients with ALS using traditional BPAP ventilation have shown that such monitoring reduces health care utilization and hospital admissions, potentially reducing overall health care costs, in comparison with routine care.^(^ 12 ^)^ This monitoring modality could be particularly useful in patients with rapidly progressive or advanced ALS, who, like our patient, might be homebound. The ability to request and verify changes to the settings remotely (without a home visit) is an additional advantage.

Volume-targeted BPAP ST mode ventilation is a relatively new alternative to traditional NPPV for patients with respiratory failure, and we have reported its use for the first time in a patient with ALS. Additional studies are needed in order to compare the various NPPV modes, in terms of their effect on survival, quality of life, sleep quality, adherence, adequacy of ventilation, and health care utilization, in ALS patients.
