# Supplementary material for: Use of volume-targeted non-invasive bilevel positive airway pressure ventilation in a patient with amyotrophic lateral sclerosis
Source: J Bras Pneumol. 2014 Jul-Aug;40(4):443–7. doi: 10.1590/S1806-37132014000400013 (PMC4201176; doi:10.1590/S1806-37132014000400013)
Supplement: Translation

# Translation: Utilização de ventilação não invasiva com dois níveis de pressão positiva nas vias aéreas e volume alvo em paciente com esclerose lateral amiotrófica*

**Authors:** Montserrat Diaz-Abad, John Edward Brown

**Affiliations:** Centro de Distúrbios do Sono, Universidade de Maryland; e Professora Assistente de Medicina. Faculdade de Medicina da Universidade de Maryland, Baltimore (MD) EUA; Centro de Distúrbios do Sono, Universidade de Maryland; e Professor Assistente de Medicina. Faculdade de Medicina da Universidade de Maryland, Baltimore (MD) EUA

**Keywords:** Esclerose amiotrófica lateral, Insuficiência respiratória, Hipoventilação, Ventilação com pressão positiva intermitente, Sono

## Abstract

A esclerose lateral amiotrófica (ELA) é uma doença neurodegenerativa progressiva. A maioria dos pacientes com ELA falece por insuficiência respiratória. Embora a ventilação não invasiva com dois níveis de pressão positiva nas vias aéreas e volume alvo tenha sido estudada em pacientes com insuficiência respiratória crônica de diferentes etiologias, sua utilização em ELA não foi relatada. Apresentamos o caso de uma mulher de 66 anos com ELA e insuficiência respiratória tratada com ventilação com dois níveis de pressão positiva e volume alvo por 15 semanas. Os dados obtidos semanalmente mostraram que a progressão da doença estava associada com aumento da fraqueza muscular respiratória, redução da respiração espontânea e maior uso de ventilação não invasiva com pressão positiva, enquanto o volume corrente e a ventilação minuto permaneceram relativamente constantes.

## Introdução

A esclerose lateral amiotrófica (ELA) é uma doença neurodegenerativa progressiva. A maioria dos pacientes com ELA morre de insuficiência respiratória em virtude de fraqueza muscular respiratória progressiva; a mediana de sobrevida após o diagnóstico é de menos de 2 anos.^(^ 1 ^)^ A ventilação não invasiva com pressão positiva (VNIPP) prolonga e melhora a qualidade de vida dos pacientes com ELA.^(^ 2 ^)^ Já se estudou o uso de ventilação não invasiva com dois níveis de pressão positiva nas vias aéreas (ventilação BPAP, do inglês *bilevel positive airway pressure*) e volume controlado, no modo espontâneo-cronometrado (E-C), com ajuste da pressão inspiratória para fornecer um volume corrente (VC) alvo estimado, em pacientes com insuficiência respiratória crônica de diferentes etiologias.^(^ 3 ^-^ 8 ^)^ No entanto, não estamos cientes de nenhum relato de uso em pacientes com ELA. 

Relatamos o caso de uma paciente com ELA de progressão rápida e insuficiência respiratória hipercápnica tratada em casa com ventilação BPAP com volume controlado no modo E-C. Além do acompanhamento clínico de rotina, foram monitorados os dados do ventilador, baixados semanalmente. 

## Relato de caso

Uma mulher de 66 anos de idade, sem história médica significativa e com índice de massa corporal de 23,4 kg/m^2^, apresentou sintomas bulbares leves e, em seguida, "pé caído" à direita. Onze meses após o início dos sintomas, recebeu diagnóstico de ELA. Na época, a CVF era de 2,22 L (79% do previsto) e a PImáx era de −28 cmH_2_O (40% do previsto). Obteve 34 pontos (de 40 possíveis) na Escala de Avaliação Funcional da ELA (EAFELA), com 10 pontos (de 12 possíveis) no componente bulbar, o que denota comprometimento leve. Obteve 8 pontos (de 21 possíveis) no Índice de Qualidade do Sono de Pittsburgh (IQSP), o que denota má qualidade do sono, e 4 (de 24 possíveis) na Escala de Sonolência de Epworth (ESE), isto é, não havia evidência de sonolência diurna excessiva. 

Aos 4 meses de acompanhamento, era evidente a acentuada progressão da doença, com agravamento dos sintomas bulbares e fadiga, assim como o eram os novos sintoma de dispneia durante a conversação, ortopneia e sono não reparador. Sua função pulmonar e seu estado funcional haviam piorado - CVF: 1,58 L (57% do previsto); PImáx: −25 cmH_2_O (36% do previsto) - e a paciente obteve 28 pontos na EAFELA, com 8 pontos no componente bulbar. A pontuação no IQSP e na ESE permaneceu relativamente inalterada (IQSP = 7 e ESE = 4). Foram feitas duas tentativas de gasometria arterial, ambas sem sucesso. Recomendamos gastrostomia e VNIPP. A paciente solicitou mais testes de confirmação antes dessas intervenções; foi então marcada para a semana seguinte uma polissonografia de noite inteira em laboratório. 

A polissonografia revelou hipoventilação durante o sono. Três semanas depois, foi realizada a titulação da ventilação BPAP em modo E-C com volume controlado (*Average Volume-Assured Pressure Support*; Philips Respironics, Murrayville, PA, EUA) por meio de uma máscara facial inteira, por escolha da paciente (Tabela 1). A paciente não tolerou o VC alvo (8 mL/kg). Portanto, a configuração final foi a seguinte: VC = 320 mL (6 mL/kg); pressão positiva inspiratória nas vias aéreas = 8-15 cmH_2_O; pressão positiva expiratória nas vias aéreas = 6 cmH_2_O (aumentada para limitação do fluxo) e tempo inspiratório = 1,5 s, com frequência de *backup* de 12 ciclos/min. Uma semana depois, a paciente retornou à clínica; apresentava agravamento contínuo dos sintomas bulbares e fraqueza, usava um andador e relatava dispneia aos mínimos esforços. Sua CVF era de 1,05 L (38% do previsto), com PImáx de −19 cmH_2_O (27% do previsto) e PaCO_2_ de 53 mmHg. Obteve 26 pontos na EAFELA (com 6 pontos no componente bulbar), 17 no IQSP e 7 na ESE. Iniciou-se VNIPP noturna com configuração de polissonografia e frequência de *backup* de 14 ciclos/min. 

**Tabela 1:** Dados do estudo do sono em paciente com esclerose lateral amiotrófica.

Parâmetro	Tipo de estudo^a^	
	PSG	AVAPS
Tempo total de sono, min	250	116
Eficiência do sono, %	55	32
Latência do sono, min	58	113
Tempo total acordada, min (%)	203 (45)	242 (65)
Estágio 1, min (%)	5 (1)	15 (4)
Estágio 2, min (%)	175 (39)	99 (27)
Estágio 3, min (%)	71 (16)	15 (4)
Sono REM, min (%)	0 (0)	0 (0)
Tempo acordada após o início do sono, min	143	31
Índice de microdespertares, eventos/h	18	6
Microdespertares espontâneos, n	67	11
Índice de movimentos periódicos dos membros, eventos/h	1	0
Índice de apneia-hipopneia, eventos/h	0	0
Média de SpO_2_ noturna, %	95	97
SpO_2_ mínima, %	93	94
ETCO_2_ basal, mmHg	46	47-54
ETCO_2_ máximo, mmHg	57	57
ETCO_2_ > 50, min	227	121
FR basal, ciclos/min	-	24
ETCO_2_ final, mmHg	-	35-45
FR final, ciclos/min	-	12-14

PSG : polissonografia

**AVAPS:** : average volume-assured pressure support (ventilação com média de pressão de suporte e volume garantido)

REM : rapid eye movement

**ETCO2:** : dióxido de carbono corrente final

a A paciente realizou ambos os estudos em decúbito, a aproximadamente 45°.

Um tubo de gastrostomia foi inserido sob orientação radiológica, e a paciente passou a ser tratada em regime de internação domiciliar, sem planos de voltar à clínica. Sete semanas após o início da VNIPP, a paciente foi contatada para ajuste da configuração com base nos sintomas e nos dados baixados (Tabela 2 e Figura 1). Decidiu fazer uma rápida visita à clínica para discutir a piora da dispneia. Apresentava dispneia leve em repouso e necessitava de uma cadeira de rodas para se mover. A VNIPP noturna, usada todas as noites, facilitava a respiração, permitindo-lhe dormir melhor e por mais tempo. Recentemente houvera um vazamento na máscara, em virtude da perda de peso da paciente. Na época, a paciente apresentava CVF de 1,01 L (36% do previsto), PImáx de −15 cmH_2_O (21% do previsto) e PaCO_2_ de 55 mmHg. A configuração do ventilador foi ajustada assim: VC = 370 mL (7 mL/kg); pressão inspiratória positiva nas vias aéreas = 10-17 cmH_2_O e tempo inspiratório = 1,2 s, com frequência de *backup* de 18 ciclos/min. Recomendamos o uso de VNIPP intermitente durante o dia e um ajuste no tamanho da máscara. O contato com a paciente (por telefone e e-mail) foi mantido, e as mudanças foram bem toleradas. Seis semanas após sua última visita, a paciente foi novamente contatada para ajuste da configuração do ventilador, mas não quis fazer mais alterações. Pouco tempo depois, morreu de insuficiência respiratória progressiva. 

**Tabela 2:** Dados do ventilador, baixados semanalmente, em paciente com esclerose lateral amiotrófica tratada com ventilação com dois níveis de pressão positiva nas vias aéreas.

Variável	Semana														
	1	2	3	4	5	6	7	8	9	10^a^	11	12	13	14	15
V_E_, L/min	4,3	4,5	4,4	4,4	4,3	4,5	5,8	5,4	5,5	6,5	7,1	6,4	5,9	6,3	6,5
VC, mL	271	267	266	259	265	259	295	273	252	310	354	346	336	336	338
Ciclos espontâneos, %	77	82	77	78	76	80	90	87	78	63	55	47	43	46	50
Uso diário, h	5,0	3,9	6,1	5,7	5,4	5,3	5,6	6,0	10,1	12,9	11,8	12,4	10,3	13,7	17,6
Uso ≥ 4 h/dia, %	71	43	100	100	86	86	86	100	100	86	100	100	43	100	100
FR, ciclos/min	19	19	20	19	20	20	22	23	25	24	22	20	20	21	22
IAH, eventos/h	15,2	19,4	25,8	21,6	19,1	23,2	5,2	13,4	22,3	26,8	7,7	15,1	11,4	5,5	12,7
Vazamento, L/min	40	40	41	38	42	38	36	39	41	38	38	36	36	35	36
PPIVA, cmH_2_O	11,9	11,9	12,9	12,8	12,5	12,6	10,9	11,8	13,5	15,0	13,8	13,5	14,2	14,0	14,5

VE : ventilação minuto

VC : volume corrente

**Ciclos espontâneos:** : ciclos respiratórios desencadeados pela paciente

Uso diário : uso do dispositivo por período de 24 h

Uso = 4 h/dia: dias em que o dispositivo foi usado durante = 4 h/ dia

IAH : índice de apneia-hipopneia

**Vazamento:** : vazamento total na máscara

**PPIVA:** : pressão positiva inspiratória nas vias aéreas

a O suporte ventilatório aumentou entre a 8ª e a 9ª semana; a 10ª semana reflete esse aumento pela primeira semana completa.

**Figura 1:** Dados do ventilador durante um período de 15 semanas em uma paciente com esclerose lateral amiotrófica tratada com ventilação com dois níveis de pressão positiva nas vias aéreas. Nota-se o aumento da duração do uso diário de ventilação com a diminuição da capacidade de desencadear ciclos respiratórios espontaneamente (isto é, o aumento da dependência dos ciclos cronometrados gerados pelo ventilador) ao longo do tempo. Como se pode observar, a ventilação minuto manteve-se relativamente constante. *O suporte ventilatório aumentou entre a 8ª e a 9ª semana; a 10ª semana reflete esse aumento pela primeira semana completa.

## Discussão

Apresentamos o caso de uma paciente com ELA tratada com ventilação BPAP com volume controlado no modo E-C durante 15 semanas em virtude de insuficiência respiratória crônica. Nessa paciente, o monitoramento semanal dos dados do ventilador mais os cuidados de rotina forneceram informações úteis para o tratamento da insuficiência respiratória. A progressão da doença associou-se a piora da fraqueza muscular respiratória, diminuição da respiração espontânea e aumento do uso de VNIPP, embora o VC e a ventilação minuto (V_E_) tenham permanecido relativamente constantes. Até onde sabemos, não há relatos de casos de ELA nos quais se tenha usado esse modo de VNIPP. 

Em pacientes com ELA, a progressão da doença é relativamente rápida, mas varia.^(^ 9 ^)^ Portanto, ajustes seriais da pressão da VNIPP podem ser necessários para compensar a diminuição da força muscular respiratória e o aumento da hipercapnia. ^(^ 10 ^)^ Um modo de VNIPP com variação de pressão inspiratória (para manter um VC alvo) em vez de pressão fixa pode reduzir a frequência de ajustes necessários ao longo do tempo em alguns pacientes. Esse recurso também pode ser benéfico em curto prazo, como durante o sono, quando os pacientes com fraqueza diafragmática estão vulneráveis à piora da hipoventilação, especialmente durante o sono *rapid eye movement*. Ambogrio et al. mostraram que, em comparação com o modo tradicional de ventilação BPAP E-C, o modo de ventilação BPAP E-C com volume controlado foi mais capaz de manter a V_E_ (por meio da manutenção do VC) durante o sono em pacientes com síndrome de hipoventilação por obesidade.^(^ 4 ^)^

O software embutido nos dispositivos de VNIPP é privado, e, na ausência de validação independente, os dados relativos a muitos parâmetros devem ser considerados indicadores de tendências, sem qualquer garantia de linearidade das estimativas.^(^ 11 ^)^ Apesar dessa limitação, os dados disponíveis podem fornecer informações valiosas para o tratamento do paciente. Estudos envolvendo o monitoramento remoto de dados de adesão à VNIPP em pacientes com ELA usando ventilação BPAP tradicional mostraram que, em comparação com os cuidados de rotina, esse monitoramento reduz a utilização de serviços de saúde e as internações hospitalares, potencialmente reduzindo os custos globais de cuidados de saúde. ^(^ 12 ^)^ Essa modalidade de monitoramento pode ser particularmente útil em pacientes com ELA avançada ou de progressão rápida, que, como nossa paciente, podem ficar impedidos de sair de casa. A capacidade de solicitar alterações na configuração e verificá-las remotamente (sem uma visita domiciliar) é uma vantagem adicional. 

A ventilação BPAP com volume controlado no modo E-C é uma alternativa relativamente nova à VNIPP tradicional para pacientes com insuficiência respiratória, e fomos os primeiros a relatar seu uso em um paciente com ELA. São necessários mais estudos a fim de comparar os vários modos de VNIPP quanto a seu efeito sobre a sobrevida, qualidade de vida, qualidade do sono, adesão, adequação da ventilação e utilização de serviços de saúde em pacientes com ELA.
